# Primary site and regional lymph node involvement are independent prognostic factors for early-stage extranodal nasal-type natural killer/T cell lymphoma

**DOI:** 10.1186/s40880-016-0096-0

**Published:** 2016-04-04

**Authors:** Shao-Qing Niu, Yong Yang, Yi-Yang Li, Ge Wen, Liang Wang, Zhi-Ming Li, Han-Yu Wang, Lu-Lu Zhang, Yun-Fei Xia, Yu-Jing Zhang

**Affiliations:** State Key Laboratory of Oncology in South China; Collaborative Innovation Center for Cancer Medicine, Sun Yat-sen University Cancer Center, Guangzhou, 510060 Guangdong P.R. China; Department of Radiation Oncology, Sun Yat-sen University Cancer Center, Guangzhou, 510060 Guangdong P.R. China; Department of Radiation Oncology, The First Affiliated Hospital of Sun Yat-sen University, Guangzhou, 510060 Guangdong P.R. China; Department of Radiation Oncology, Cancer Hospital and Institute, Peking Union Medical College (PUMC) and Chinese Academy of Medical Sciences (CAMS), Beijing, 100021 P.R. China; Department of Nuclear Medicine, The Third Affiliated Hospital of Guangzhou Medical University, Guangzhou, 510150 Guangdong P.R. China; Department of Hematological Oncology, Sun Yat-sen University Cancer Center, Guangzhou, 510060 Guangdong P.R. China; Department of Medical Oncology, Sun Yat-sen University Cancer Center, Guangzhou, 510060 Guangdong P.R. China

**Keywords:** Extranodal natural killer/T-cell lymphoma (ENKTCL), Nasal cavity, Lymph node, Prognosis, Nomogram

## Abstract

**Background:**

Nasal-type extranodal natural killer/T-cell lymphoma (ENKTCL) originates primarily in the nasal cavity or extra-nasal sites within the upper aerodigestive tract. However, it is unclear whether the primary site can serve as an independent prognostic factor or whether the varying clinical outcomes observed with different primary sites can be attributed merely to their propensities of regional lymph node involvement. The aim of this study was to investigate the prognostic implications of the primary site and regional lymph node involvement in patients with early-stage nasal-type ENKTCL.

**Methods:**

To develop a nomogram, we reviewed the clinical data of 215 consecutively diagnosed patients with early-stage nasal-type ENKTCL who were treated in Sun Yat-sen University Cancer Center with chemotherapy and radiotherapy between 2000 and 2011. The predictive accuracy and discriminative ability of the nomogram were determined using a concordance index (C-index) and calibration curve.

**Results:**

The 5-year overall survival (OS) and progression-free survival (PFS) rates of patients with nasal ENKTCL were higher than those of patients with extra-nasal ENKTCL (OS: 68.2% vs. 46.0%, *P* = 0.030; PFS: 53.4% vs. 26.6%, *P* = 0.010). The 5-year OS and PFS rates of patients with Ann Arbor stage IE ENKTCL were higher than those of patients with Ann Arbor stage IIE ENKTCL (OS: 66.3% vs. 59.2%, *P* = 0.003; PFS: 51.4% vs. 40.3%, *P* = 0.009). Multivariate analysis showed that age >60 years, ECOG performance status score ≥2, elevated lactate dehydrogenase (LDH) level, extra-nasal primary site, and regional lymph node involvement were significantly associated with lower 5-year OS rate; age >60 years, elevated LDH level, extra-nasal primary site, and regional lymph node involvement were significantly associated with lower 5-year PFS rate. The nomogram included the primary site and regional lymph node involvement based on multivariate analysis. The calibration curve showed good agreement between the predicted and actual 5-year OS and PFS rates, and the C-indexes of the nomogram for the OS and PFS rates were 0.697 and 0.634, respectively.

**Conclusions:**

The primary site and regional lymph node involvement are independent prognostic factors for early-stage ENKTCL treated with chemotherapy followed by definitive radiotherapy.

## Background

According to the World Health Organization (WHO) classification, nasal-type extranodal natural killer (NK)/T-cell lymphoma (ENKTCL) is a distinct histopathologic type of non-Hodgkin lymphoma [1]. Most ENKTCL originates in the upper aerodigestive tract (UADT), which includes the nasal cavity, nasopharynx, paranasal sinuses, tonsils, hypopharynx, and larynx [2–4]. Most published studies reported the use of anthracycline-based chemotherapy; however, unsatisfactory results were obtained [5]. Therefore, L-asparaginase-based chemotherapy regimens are currently being investigated [6–10]. Although the optimal treatment remains unknown, definitive radiotherapy used upfront or after short-course chemotherapy is considered the standard treatment for early-stage ENKTCL [11–15]. Previous studies found that regional lymph node involvement is more common in patients with ENKTCL that originates in the Waldeyer’s ring or has involvement in the Waldeyer’s ring, and this variance can contribute to differences in long-term survival [16–19]. These studies considered the primary site of the disease to be a significant prognostic factor. However, it is unclear whether the primary site can serve as an independent prognostic factor or whether it merely acts through its interaction with regional lymph node involvement or disease stage.

 Nomogram is a graphical statistical prediction model that provides a clearly decipherable prediction and can be used to determine the relationship between multiple prognostic factors [20–22]. The performance (discrimination and calibration) of a nomogram has been rigorously assessed, although no study to date has reported the use of nomogram prediction for patients with early-stage UADT-ENKTCL. Hence, the goal of our study was to use a nomogram to determine whether the primary site and regional lymph node involvement are independent prognostic factors for this lymphoma.

## Patients and methods

### Patient selection

This study included 215 patients diagnosed with Ann Arbor stages IE–IIE UADT-ENKTCL between January 2000 and December 2011 at Sun Yat-sen University Cancer Center. The diagnostic criteria were based on the WHO classification of tumors of hematopoietic and lymphoid tissues [1]. Based on a patient’s primary symptoms and if the majority of the lymphoma bulk presented in the nasal cavity, with or without extension to adjacent structures, the disease was considered primary nasal cavity ENKTCL (nasal ENKTCL); if the majority of the lymphoma bulk presented in extra-nasal sites, the disease was considered extra-nasal ENKTCL. Local tumor invasiveness (LTI) was defined as extension of the primary site, bone invasion, or perforation or invasion of the skin [23]. The diseases of all patients were staged according to the Ann Arbor staging system. The clinical evaluation of patients included a history and physical examination, complete blood count, liver and renal function tests, serum lactate dehydrogenase (LDH) level detection, chest radiograph, computed tomography of the chest and abdomen/pelvis, bone marrow aspiration and/or biopsy, and computed tomography and/or magnetic resonance imaging of the head and neck.

### Treatment

All patients received radiotherapy: 4 patients received radiotherapy alone, and 211 patients received a combination of radiotherapy and chemotherapy (202 received chemotherapy followed by radiotherapy, and 9 received radiotherapy followed by chemotherapy). The chemotherapy regimen was mostly anthracycline-based, including a cyclophosphamide, doxorubicin, vincristine, and prednisolone (CHOP) or CHOP-like regimen in 175 patients and an L-asparaginase-based regimen in 31 patients; 5 patients received other chemotherapy regimens. The median number of chemotherapy cycles before radiotherapy was 3 cycles (range, 1–9 cycles). Radiotherapy was administered with a 6-MV or 8-MV linear accelerator. The median dose was 55 Gy (36–70 Gy) with conventional fractionation; 181 patients (84.2%) received radiotherapy with a dose range of 46–70 Gy (median, 56 Gy).

### Construction of the nomogram

The variables for the nomogram were selected based on variables that have been previously shown to be associated with survival. These variables included age, eastern cooperative oncology group (ECOG) performance status (PS), LTI, “B” symptoms, LDH levels, regional lymph node involvement, and the primary site of disease. A multivariable Cox proportional hazards model was used to estimate the 5-year overall survival (OS) and progression-free survival (PFS) rates for each variable. Discrimination and calibration powers were examined using a concordance index (C-index) and a calibration curve.

### Statistical analysis

OS was measured from the start of the initial treatment until the time of death from any cause or until the last follow-up. PFS was measured from the start of the initial treatment until the first loco-regional or distant progression, relapse, last follow-up, or death. A survival curve was constructed using the Kaplan–Meier method, and the groups were compared using the log-rank test. A multivariable Cox proportional hazards model was used to confirm independent prognostic factors, and the nomogram was constructed based on the Cox model parameters. The selection of the final model was determined by a backward step-down selection process. The C-index was estimated by analyzing the area under the curve of the receiver operating characteristic (ROC) curve to calculate an unbiased measure of the validity of the model. Statistical analyses were performed using IBM SPSS Statistics software, version 20.0 (IBM, Chicago, IL, USA). *P* values <0.05 were considered statistically significant.

## Results

### Patient characteristics

In all 215 patients included in this study, the ratio of men to women was 1.9:1, and only 27 patients (12.6%) were 60 years of age or older (range, 9–80 years; median, 42 years). Of the 215 patients, 67 (31.2%) had regional node involvement (or Ann Arbor stage IIE disease), and 87 (40.5%) presented with systemic symptoms; 207 (96.3%) had an ambulatory ECOG PS of 0–1, and 43 (20.0%) had an elevated LDH level; 126 (58.6%) presented with LTI. Similar clinical characteristics were observed in patients whose primary tumor was located in the nasal cavity (nasal ENKTCL) and in extra-nasal sites (extra-nasal ENKTCL). The baseline characteristics of the cohort are detailed in Table 1.

**Table 1:** Clinicopathologic characteristics of patients with early-stage UADT-ENKTCL

Characteristic	All patients (*n* = 215)	Primary site		*P*
		Nasal (*n* = 170)	Extra-nasal (*n* = 45)	
Sex				0.594
Men	141 (65.6)	113 (66.5)	28 (62.2)	
Women	74 (34.4)	57 (33.5)	17 (37.8)	
Age (years)				0.404
≤60	188 (87.4)	147 (86.5)	41 (91.1)	
>60	27 (12.6)	23 (13.5)	4 (8.9)	
Regional lymph node involvement				0.150
Yes	67 (31.2)	49 (28.8)	18 (40.0)	
No	148 (68.8)	121 (71.2)	27 (60.0)	
“B” symptoms				0.048
Yes	87 (40.5)	63 (37.1)	24 (53.3)	
No	128 (59.5)	107 (62.9)	21 (46.7)	
Elevated LDH				0.675
Yes	43 (20.0)	35 (20.6)	8 (17.8)	
No	172 (80.0)	135 (79.4)	37 (82.2)	
LTI				0.137
Yes	126 (58.6)	104 (61.2)	22 (48.9)	
No	89 (41.4)	66 (38.8)	23 (51.1)	
ECOG score				0.773
0–1	207 (96.3)	164 (96.5)	43 (95.6)	
≥2	8 (3.7)	6 (3.5)	2 (4.4)	

All values are presented as the number of patients followed by the percentages in the parentheses

*UADT-ENKTCL* upper aerodigestive tract-extranodal natural killer/T-cell lymphoma, *LDH* lactate dehydrogenase, *LTI* local tumor invasiveness, *ECOG* eastern cooperative oncology group

### Survival and univariate analysis

**Fig. 1:**
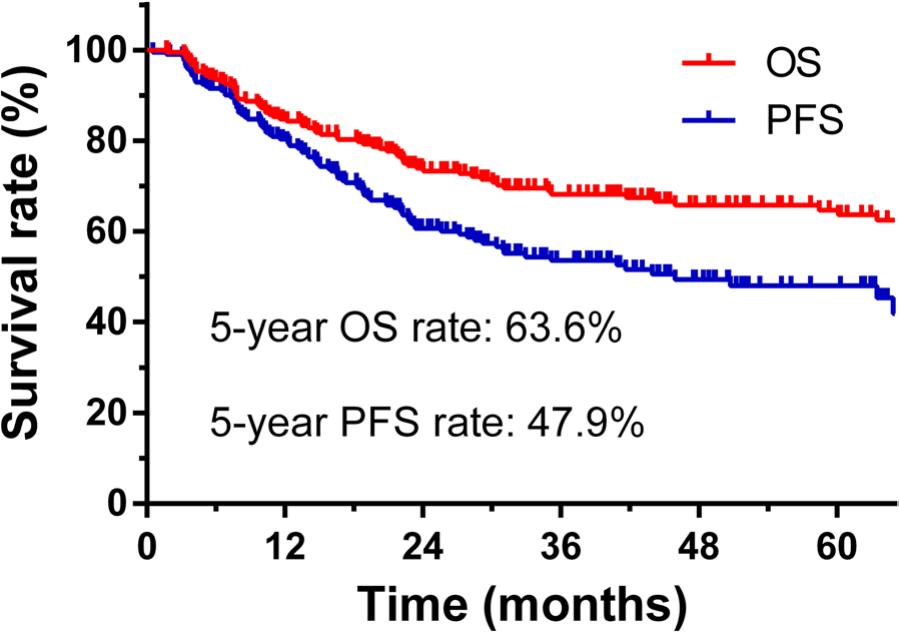
Overall survival (OS) and progression-free survival (PFS) of 215 patients with early-stage nasal-type extranodal natural killer/T-cell lymphoma (ENKTCL)

**Fig. 2:**
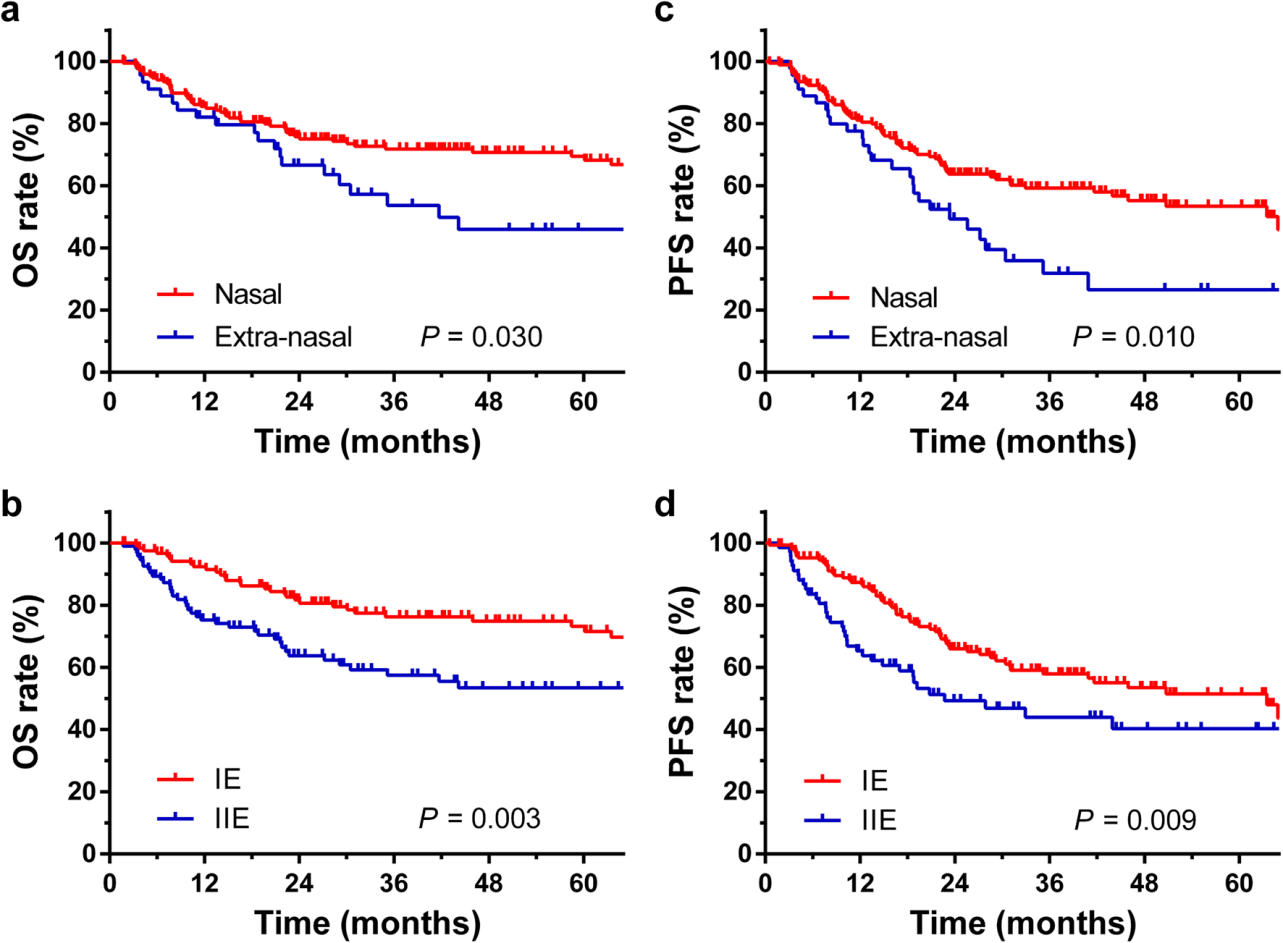
Kaplan-Meier OS and PFS *curves* for patients with ENKTCL categorized by primary site and regional lymph node involvement. **a** OS *curves* for patients with nasal and extra-nasal disease; **b** OS *curves* for patients with Ann Arbor stage IIE or IE disease (i.e., with or without regional lymph node involvement); **c** PFS *curves* for patients with nasal and extra-nasal disease; **d** PFS *curves* for patients with Ann Arbor stage IE or IIE disease

After a median follow-up of 47.7 months, 68 patients had died. The 5-year OS and PFS rates were 63.6% and 47.9%, respectively (Fig. 1). According to the univariate analysis results, the following variables were associated with the OS rate: age (>60 vs. ≤60 years, 38.9% vs. 66.7%, *P* = 0.001), performance status (ECOG score ≥2 vs. <2, 25.0% vs. 65.4%, *P* = 0.008), LDH level (increased level vs. normal, 48.7% vs. 67.3%, *P* = 0.002), extra-nasal primary site of lymphoma (yes vs. no, 46.0% vs. 68.2%, *P* = 0.030, Fig. 2a), and regional lymph node involvement (yes vs. no, 59.2% vs. 66.3%, *P* = 0.003, Fig. 2b). The following variables were associated with the PFS rate: LDH level (increased level vs. normal, 35.6% vs. 51.2%, *P* = 0.006), extra-nasal primary site of lymphoma (yes vs. no, 26.6% vs. 53.4%, *P* = 0.010, Fig. 2c), and regional lymph node involvement (yes vs. no, 40.3% vs. 51.4%, *P* = 0.009, Fig. 2d).

**Fig. 3:**
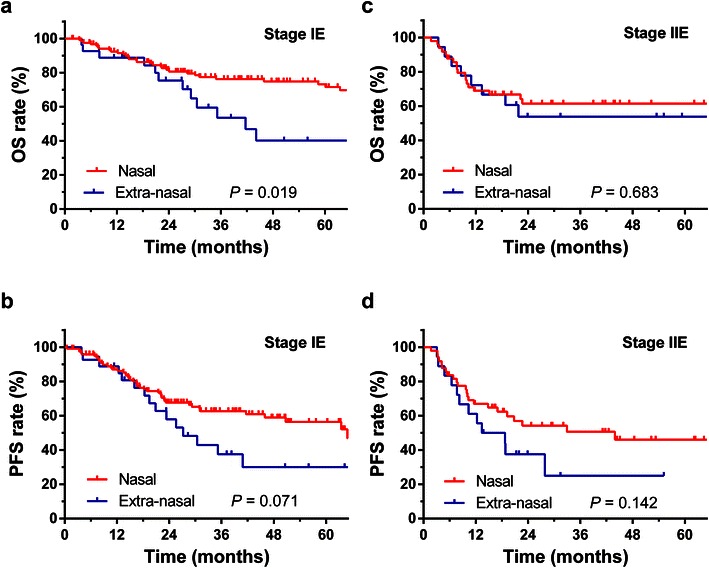
Survival *curves* for patients with nasal and extra-nasal ENKTCL at Ann Arbor stage IE and IIE. **a** OS for patients with nasal and extra-nasal disease at Ann Arbor stage IE; **b** PFS for patients with nasal and extra-nasal disease at Ann Arbor stage IE; **c** OS for patients with nasal and extra-nasal disease at Ann Arbor stage IIE; **d** PFS for patients with nasal and extra-nasal disease at Ann Arbor stage IIE

For Ann Arbor stage IE patients, the 5-year OS rate was 71.5% for those with nasal ENKTCL and 40.2% for those with extra-nasal ENKTCL (*P* = 0.019, Fig. 3a), with a trend toward a higher PFS rate (56.4% vs. 30.0%, *P* = 0.071, Fig. 3b). For Ann Arbor stage IIE patients, the 5-year OS rate was 61.4% for those with nasal ENKTCL and 53.8% for those with extra-nasal ENKTCL (*P* = 0.683, Fig. 3c); the 5-year PFS rate was 46.0% for patients with nasal ENKTCL and 25.0% for patients with extra-nasal ENKTCL (*P* = 0.142, Fig. 3d).

### Prognostic nomogram for OS and PFS

A multivariate analysis was conducted using Cox proportional hazards regression, and this Cox model was the basis for the nomogram. The multivariate analysis results for the 215 patients with complete data are presented in Table 2. Age >60 years (hazard ratio [HR] = 3.365, *P* < 0.001), ECOG score ≥2 (HR = 2.439, *P* = 0.040), elevated LDH level (HR = 2.422, *P* = 0.001), extra-nasal primary site (HR = 1.860, *P* = 0.022), and regional lymph node involvement (HR, 1.796; *P* = 0.024) were significantly associated with a lower 5-year OS rate. Age >60 years (HR = 1.720, *P* = 0.057), elevated LDH level (HR = 1.942, *P* = 0.005), extra-nasal primary site (HR = 1.857, *P* = 0.007), and regional lymph node involvement (HR = 1.716, *P* = 0.014) were significantly associated with a lower 5-year PFS rate.

**Table 2:** Multivariate analysis of 215 patients with early-stage UADT-ENKTCL

Variable	Overall survival			Progression-free survival		
	HR	95% CI	*P*	HR	95% CI	*P*
Primary site (extra-nasal vs. nasal)	1.860	1.092–3.169	0.022	1.857	1.182–2.916	0.007
Regional lymph node involvement (yes vs. no)	1.796	1.080–2.986	0.024	1.716	1.116–2.637	0.014
Age (>60 vs. ≤60 years)	3.365	1.867–6.066	<0.001	1.720	0.984–3.006	0.057
Elevated LDH level (yes vs. no)	2.422	1.419–4.132	0.001	1.942	1.218–3.093	0.005
ECOG score (≥2 vs. 0–1)	2.439	1.410–5.690	0.040	–	–	–

*HR* hazard ratio, *CI* confidence interval, *UADT-ENKTCL* upper aerodigestive tract-extranodal natural killer/T-cell lymphoma, *LDH* lactate dehydrogenase, *LTI* local tumor invasiveness, *ECOG* eastern cooperative oncology group

**Fig. 4:**
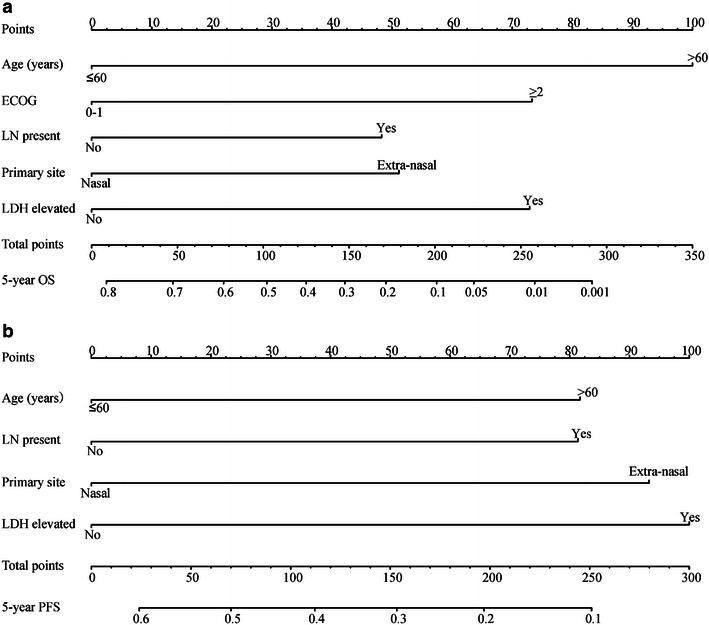
A nomogram for patients with early-stage upper aerodigestive tract natural killer/T-cell lymphoma. To use the nomogram, an individual patient’s value is located on each variable axis, and a line is drawn upward to determine the number of points received for each variable value. The sum of these numbers is located on the total points axis, and a line is drawn downward to the survival axes to determine the likelihood of 5-year OS (**a**) and PFS (**b**) rates. *Site* primary site of the lymphoma, *LDH* pretreatment level of serum lactate dehydrogenase, *ECOG* eastern cooperative oncology group, *LN* regional lymph node

**Fig. 5:**
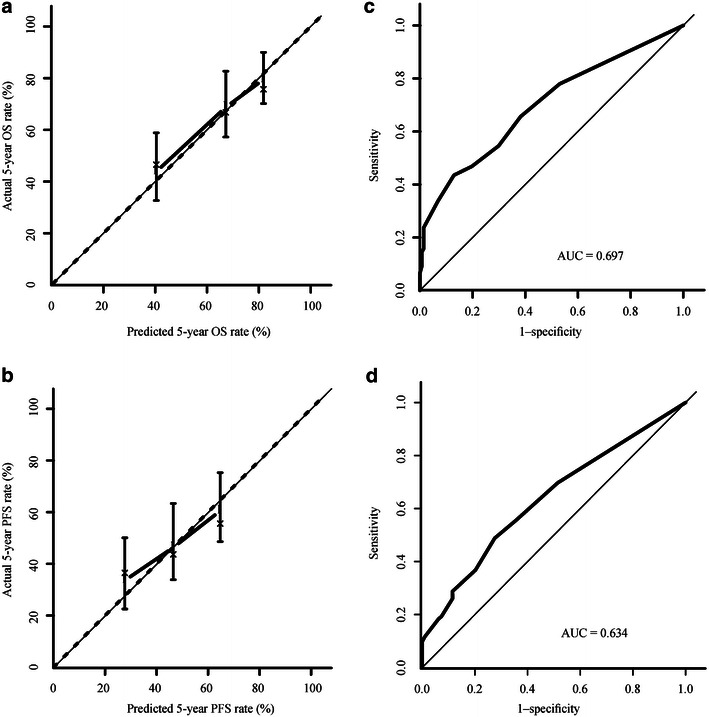
Internal validation of the nomogram used to predict OS and PFS in patients with upper aerodigestive tract nasal-type extranodal natural killer/T-cell lymphoma. Discrimination and Calibration: Calibration plot for predicting 5-year OS rate (**a**) and 5-year PFS rate (**b**). The areas under the receiver operating characteristic curve are 0.697 for 5-year OS rate (**c**) and 0.634 for 5-year PFS rate (**d**). The nomogram-predicted survival is plotted on the *X axis*; the actual survival is plotted on the *Y axis*. *AUC* area under the curve

The prognostic nomogram that includes all significant independent factors for the 5-year OS and PFS rates is presented in Fig. 4a and b. Based on this nomogram, an individual patient’s value for each variable was scored by locating the corresponding position on the variable scale and drawing a vertical line to determine the corresponding points. The total points were then tallied, and a vertical line was drawn through the survival scales to calculate a 5-year survival rate. The calibration plot for the 5-year survival rate showed an optimal agreement between the prediction obtained with the nomogram and the actual observation (Fig. 5a, b). The C-indexes for OS and PFS prediction by the nomogram were 0.697 and 0.634, respectively (Fig. 5c, d).

## Discussion

Our results confirm that the primary site and regional lymph node involvement were both significant prognostic factors in patients with early-stage UADT-ENKTCL who received definitive radiotherapy that was administered mainly after chemotherapy. Patients with Ann Arbor stage IE disease or nasal ENKTCL had a favorable prognosis, with the 5-year OS and PFS rates of approximately 70%–50%, respectively.

Patients with ENKTCL at different sites have different clinical presentations. The UADT is the most common involved site of NK/T-cell lymphoma. Most patients with UADT-ENKTCL (60%–80%) present with early-stage disease, although only a small proportion of patients (10%–40%) show regional lymph node involvement (Ann Arbor stage IIE) [2, 22–25]. However, compared with patients with nasal ENKTCL, patients with extra-nasal UADT-ENKTCL are more likely to present with regional lymph node involvement, and the involvement of regional lymph nodes has been shown to be associated with poor outcomes [17–19, 24]. Patients with extra-nasal UADT-ENKTCL also have higher LDH levels, more bulky disease, and a worse performance status. All these adverse clinical features may contribute to their poor survival [18]. Previous studies that included patients with advanced-stage (stages IIIE–IVE) diseases and some patients who received only chemotherapy have suggested that, based on univariate analysis, the primary site of the tumor may be a prognostic factor [16, 17, 19, 25]. Therefore, we initially confirmed the primary site as an independent prognostic factor for patients with early-stage (stages I–II) UADT-ENKTCL using univariate and multivariate analyses. Age, performance status, LDH level, and local regional lymph node involvement were also confirmed as independent prognostic factors.

For studies with time-to-event data, a Cox proportional hazards model has been used as a standard method. However, the performance (discrimination and calibration) of this model has generally not been rigorously assessed, and it is not an ideal tool for the prediction of an individual patient’s outcome. The nomogram used in our study, which performed well in predicting long-term survival, clearly suggested primary site and locoregional lymph node involvement as independent prognostic factors for both OS and PFS in patients with early-stage (stages I–II) UADT-ENKTCL; this prediction was supported by validation (the C-indexes for OS and PFS were 0.697 and 0.634, respectively) and a calibration curve. Furthermore, the nomogram is a practical, predictive tool for determining prognosis, owing to the fact that the clinical variables used in the model are readily available to any physician.

In recent years, radiotherapy has been considered the most effective treatment for early-stage ENKTCL, resulting in a 50%–90% long-term survival rate [26–28]. However, radiotherapy may result in different outcomes depending on whether it is used upfront or whether it is delayed. Li and colleagues [17, 19] reported the clinical features and outcomes of 95 patients with early-stage Waldeyer’s ring ENKTCL (WR-ENKTCL) compared with those of 145 patients with early-stage nasal ENKTCL; their results indicated that patients with Ann Arbor stage IIE WR-ENKTCL had higher 5-year OS and PFS rates than patients with nasal ENKTCL at the same stage. In contrast, we found that nasal ENKTCL patients had higher 5-year OS and PFS rates than extra-nasal ENKTCL patients, and patients with Ann Arbor stage IE nasal ENKTCL had a higher 5-year OS rate than patients with extra-nasal ENKTCL, although the 5-year OS rate between the two groups was similar for patients with Ann Arbor stage IIE disease. These differences may be related to the initial treatment. In our study, most patients (94%) received chemotherapy first, whereas in Li and colleagues’ study approximately 60% of patients received radiotherapy as their first treatment [19]. For patients in our study, the 5-year OS and PFS rates were 63.6% and 47.9%, respectively, which were lower than the rates in Li and colleagues’ study [19]. Because patients with extra-nasal ENKTCL are more likely to have Ann Arbor stage IIE disease, a possible explanation for this result may be that delayed radiotherapy leads to inferior tumor control with ineffective initial chemotherapy, and this tendency may be more obvious in patients with extra-nasal ENKTCL because of the propensity for lymph node metastasis. Thus, we conclude that when patients receive initial chemotherapy followed by radiotherapy, patients with extra-nasal ENKTCL have lower long-term survival rate compared with patients with nasal ENKTCL.

The main limitations of our study were its retrospective nature and its small sample size because of the lower incidence of this tumor in South China. Some potential prognostic biomarkers reported in recent years, such as Ki-67 scores and circulating Epstein-Barr virus DNA, were not included as variables in the nomogram because many biological or molecular markers are not widely available to physicians during early-phase diagnosis and treatment. Furthermore, because ENKTCL that originates at sites outside of the UADT (e.g., the skin and gastrointestinal wall) is rare, patients with these disease characteristics were not included in our study.

In conclusion, as demonstrated by a nomogram in our group of 215 patients, primary site and regional lymph node involvement have clear independent prognostic values in early-stage UADT-ENKTCL. In this group of patients, those with early-stage nasal ENKTCL may survive longer than those with extra-nasal ENKTCL at the same stage, even though most patients were initially treated with chemotherapy followed by definitive radiotherapy. It is still unclear whether radiotherapy that was initially provided and a more effective chemotherapy regimen could alter this prognostic pattern.
